# Protective effect of resveratrol against caspase 3 activation in primary mouse fibroblasts

**DOI:** 10.3325/cmj.2015.56.78

**Published:** 2015-04

**Authors:** Zsófia Ulakcsai, Fruzsina Bagaméry, István Vincze, Éva Szökő, Tamás Tábi

**Affiliations:** Department of Pharmacodynamics, Semmelweis University, Budapest, Hungary

## Abstract

**Aim:**

To study the effect of resveratrol on survival and caspase 3 activation in non-transformed cells after serum deprivation.

**Methods:**

Apoptosis was induced by serum deprivation in primary mouse embryonic fibroblasts. Caspase 3 activation and lactate dehydrogenase release were assayed as cell viability measure by using their fluorogenic substrates. The involvement of PI3K, ERK, JNK, p38, and SIRT1 signaling pathways was also examined.

**Results:**

Serum deprivation of primary fibroblasts induced significant activation of caspase 3 within 3 hours and reduced cell viability after 24 hours. Resveratrol dose-dependently prevented caspase activation and improved cell viability with 50% inhibitory concentration (IC_50_) = 66.3 ± 13.81 µM. It also reduced the already up-regulated caspase 3 activity when it was added to the cell culture medium after 3 hour serum deprivation, suggesting its rescue effect. Among the major signaling pathways, p38 kinase was critical for the protective effect of resveratrol which was abolished completely in the presence of p38 inhibitor.

**Conclusion:**

Resveratrol showed protective effect against cell death in a rather high dose. Involvement of p38 kinase in this effect suggests the role of mild stress in its cytoprotective action. Furthermore due to its rescue effect, resveratrol may be used not only for prevention, but also treatment of age-related degenerative diseases, but in the higher dose than consumed in conventional diet.

Age-related degenerative diseases pose enormous challenges both for individuals and society in terms of life quality and economic burden. Since age-related neurodegenerative and cardiovascular diseases develop mainly as a result of cell impairments, it is crucial to find agents that prevent and abolish cell damage and death. Resveratrol (3,5,4'-tihydroxy-*trans*-stilbene) is a widely investigated phytoalexin compound, which can be found in numerous plants, mainly in the skin and seeds of red grapes (1). It was reported to possess multiple pharmacological properties including antiaging (2), antioxidative, anti-inflammatory (3), anticarcinogenic (4), and neuro- and cardioprotective effects (5). However, in the literature its rather contradictory properties, ie, cytoprotective and proapoptotic, were reported (6). The cause of opposite effects may lie in different cell types, cell states, and the duration or dosage of treatment used in the various models (7). Characteristically, resveratrol has an opposite impact on apoptosis in non-transformed and transformed cells (8,9). The targets of resveratrol and the mechanisms governing its effects are currently unclear. It was reported to affect different metabolic and signaling pathways, exhibit pro- or antioxidative activities, and modify the functions of several transcription factors and cofactors (10).

Since resveratrol might differently affect apoptotic process of tumorigenic and non-transformed normal cells, the aim of this study was to investigate its effect on the death of non-transformed cells as a potential lead compound for research of cytoprotective medications. We used primary mouse embryonic fibroblasts as an easily available non-transformed cell culture model. In order to evaluate its cytoprotective effect, caspase 3 activation was examined following serum deprivation as a model of insufficient availability of trophic factors. The specific background mechanisms, involvement of the PI3K, ERK, JNK, p38, and SIRT1 signaling pathways were also determined.

## Materials and methods

### Reagents and animals

Resveratrol, the inhibitors of kinases (SB202190 for p38 MAPK, SP600125 for JNK, PD184352 for ERK, wortmannin for PI3K) and SIRT1 (EX-527), caspase 3 activity assay kit using fluorogenic caspase 3 substrate (Ac-DEVD-AMC), buffer components and N-acetylcysteine were purchased from Sigma-Aldrich (St. Louis, MO, USA). Non-selective caspase inhibitor (Ac-VAD-CMK) was obtained from AnaSpec (Fermont, CA, USA) and CytoxOne lactate dehydrogenase release kit from Promega (Fitchburg, WI, USA). Cell culture mediums and fetal bovine serum were supplied by GE Healthcare (Little Chalfont, UK) and Life Technologies (Carlsbad, CA, USA), respectively. Test compounds were dissolved in DMSO and used in cell culture medium to provide 0.5% final DMSO concentration. Control cells were treated with the same concentration of DMSO.

Pregnant NMRI mice were supplied by Toxicoop, Gödöllő, Hungary. All animal procedures were approved by the ethics committee of the Semmelweis University (22.1/606/001/2010, February 5, 2010) and were in accordance with the EU Council directives on laboratory animals (86/609/EEC).

### Cell culture conditions and assay for caspase 3 activity and lactate dehydrogenase release

Mouse embryonic fibroblast culture was established according to CSH protocol (11). Cells were maintained in DMEM supplemented with 10% fetal bovine serum and used between passage 3 and 7. One day before the experiment cells were seeded to 6 cm Petri dishes (3 × 10^5^ cells/dish). Twenty-four hours later fetal bovine serum was withdrawn from the cell culture medium to induce cell death. Resveratrol treatment was initiated simultaneously with serum deprivation. When the rescue effect of resveratrol was investigated, resveratrol was added to the cell culture medium after 3-hour serum deprivation. Inhibitors of various signaling pathways were applied simultaneously with serum deprivation and/or resveratrol treatment.

For caspase activity assay after specified treatment periods (3, 4.5, 6 hours), cells were rinsed with PBS and harvested by trypsin-EDTA, and cytosol extract was prepared by hypotonic lysis with 0.6% Nonidet P40 according to Andrews and Faller (12). In order to evaluate direct caspase inhibitory effect of resveratrol, resveratrol was added directly to cytosol extract of serum-deprived fibroblasts immediately before measuring caspase 3 activity. Ac-VAD-CMK, a non-selective direct caspase inhibitor, was used in 20 µM concentration as positive control. Caspase 3 activity and lactate dehydrogenase release were measured by commercially available kits according to the manufacturer instructions. Caspase 3 activity is shown as nanomol substrate cleaved by miligram protein in 3 hours.

### Statistical analysis

Data were expressed as mean ± standard deviation. Comparisons were made by paired *t* test. *P* < 0.05 was considered statistically significant. Data were analyzed by Microsoft Excel 2010 (Redmond, WA, USA).

## Results

### Resveratrol dose-dependently prevented serum deprivation-induced caspase 3 activation in primary mouse embryonic fibroblasts

**Figure 1:**
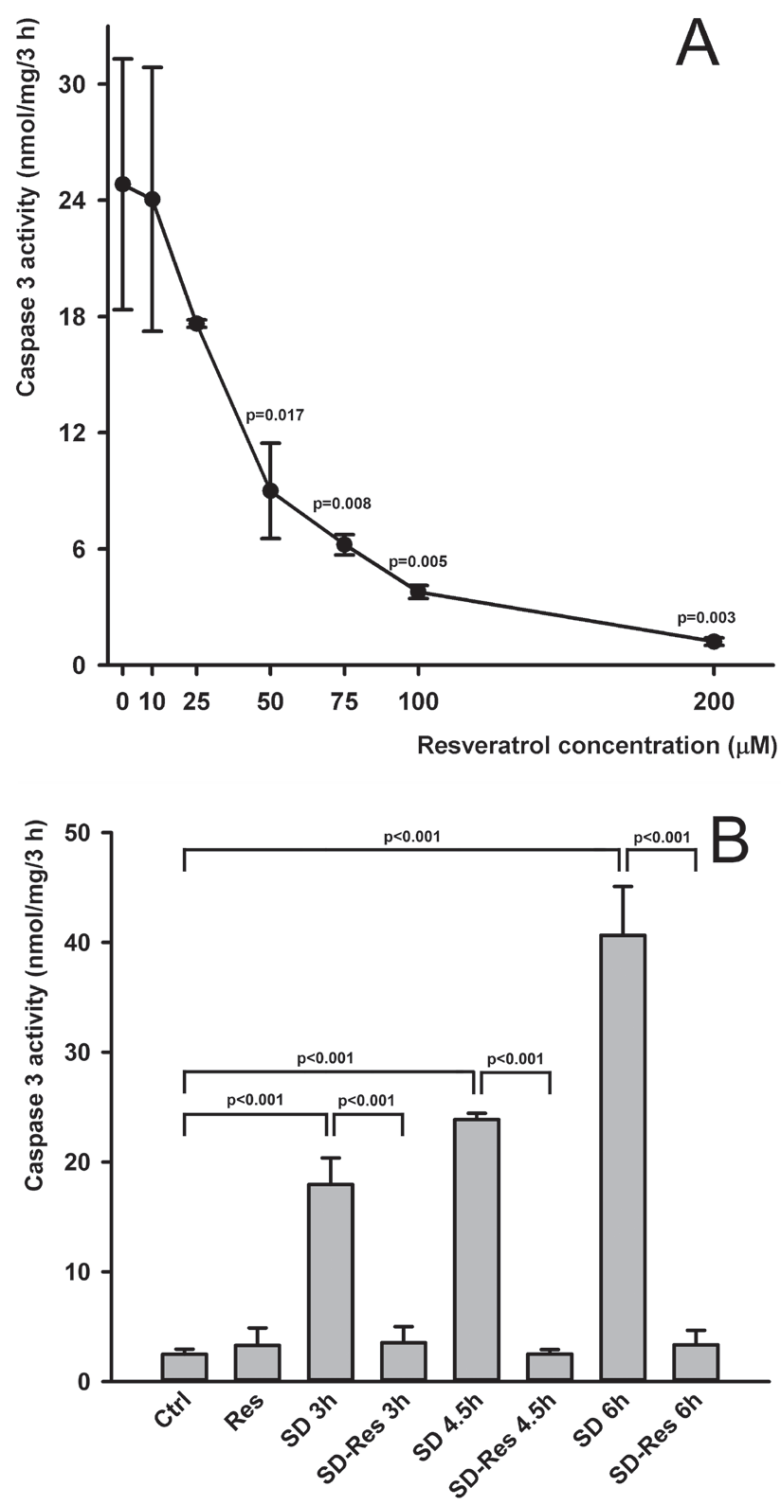
Resveratrol dose-dependently prevented caspase 3 activation after 3 h serum deprivation. Control value of caspase 3 activity in serum supported cells: 1.76 ± 0.097 nmol/mg/3 h (**A**). 200 µM of resveratrol prevented caspase 3 activation after 3, 4.5, and 6 h serum deprivation (**B**).

**Figure 2:**
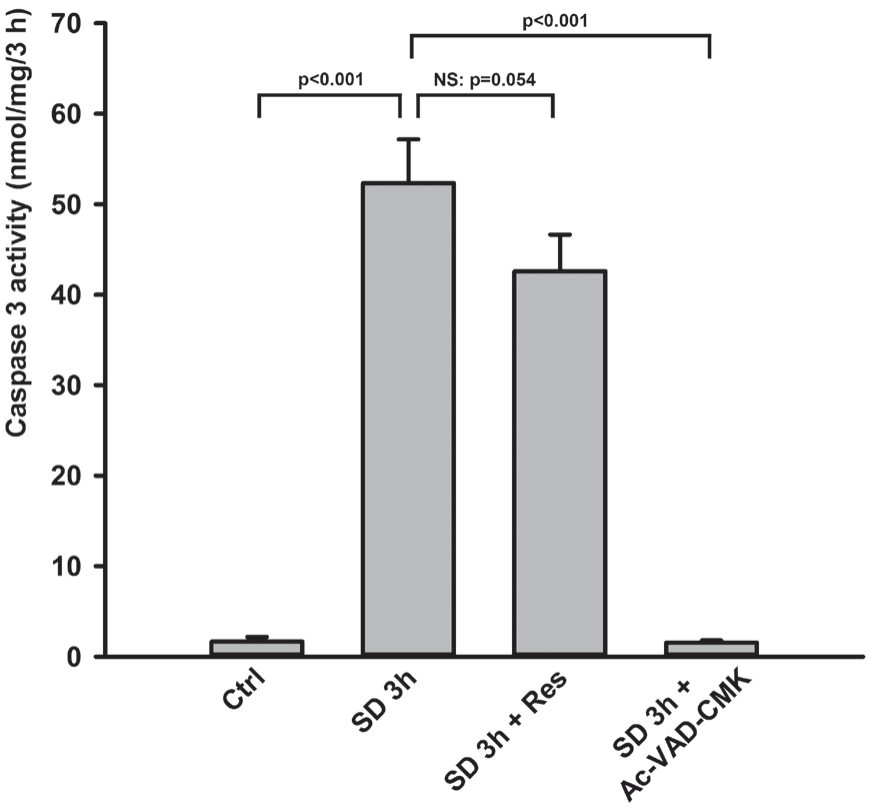
Resveratrol showed no direct caspase 3 inhibitory effect. When 200 µM of resveratrol was added to cytosol extract of serum-deprived fibroblast during caspase 3 activity measurement, it did not significantly reduce caspase 3 activity. A known direct caspase inhibitor, Ac-VAD-CMK, was used as positive control in 20 µM concentration.

Primary mouse fibroblasts were exposed to serum deprivation, which after 3-6 hours induced significant caspase 3 activation (*P* < 0.001). In order to evaluate the protective effect of resveratrol, the cells were treated with several concentrations (10, 25, 50, 75, 100, 200 µM) of resveratrol simultaneously with serum deprivation. Resveratrol prevented caspase 3 activation in a dose-dependent manner, with 50% inhibitory concentration (IC_50_) = 66.3 ± 13.81 µM. Caspase 3 activation following 3 hour serum deprivation was completely inhibited at 200 µM resveratrol concentration (Figure 1A), and thus this level was used in the further experiments. This protective effect was also obtained after up to 6 hours of serum deprivation (Figure 1B). To verify whether resveratrol regulates the cellular response or directly interacts with caspase 3, resveratrol was added directly to the cytosol extract rather than to cell culture medium. Resveratrol showed no direct caspase inhibitory effect, although the known direct inhibitor Ac-VAD-CMK, used as positive control, completely blocked caspase 3 activity (Figure 2).

### Resveratrol exhibited rescue effect on serum deprivation-induced caspase 3 activation

**Figure 3:**
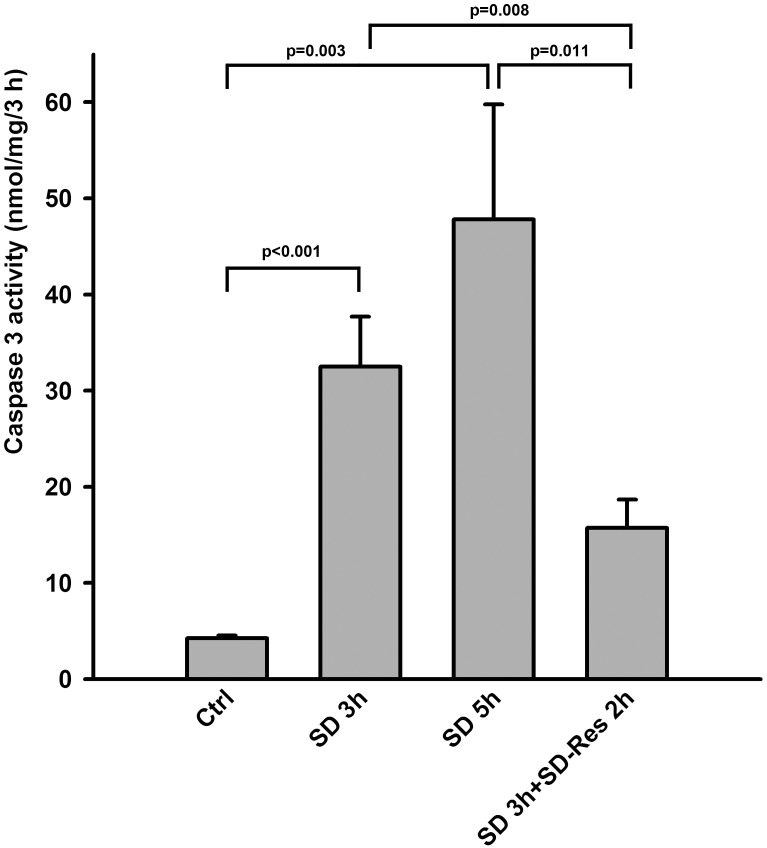
Resveratrol showed rescue effect on caspase 3 activation. Following 3 h of serum deprivation, 200 µM resveratrol was supplemented for an additional 2 h.

We further investigated whether resveratrol reduced the already up-regulated caspase 3 activity. Primary fibroblasts were exposed to serum deprivation for 3 hours, after which the culture medium was supplemented with 200 µM resveratrol for an additional 2 hours. Resveratrol significantly reduced the already activated caspase 3. It prevented not only its further increase but also reduced it to a level below that observed after 3-hour serum deprivation. These experiments indicate that resveratrol may have both protective and rescue effect on cells (Figure 3).

### Resveratrol reduced lactate dehydrogenase release induced by serum deprivation

**Figure 4:**
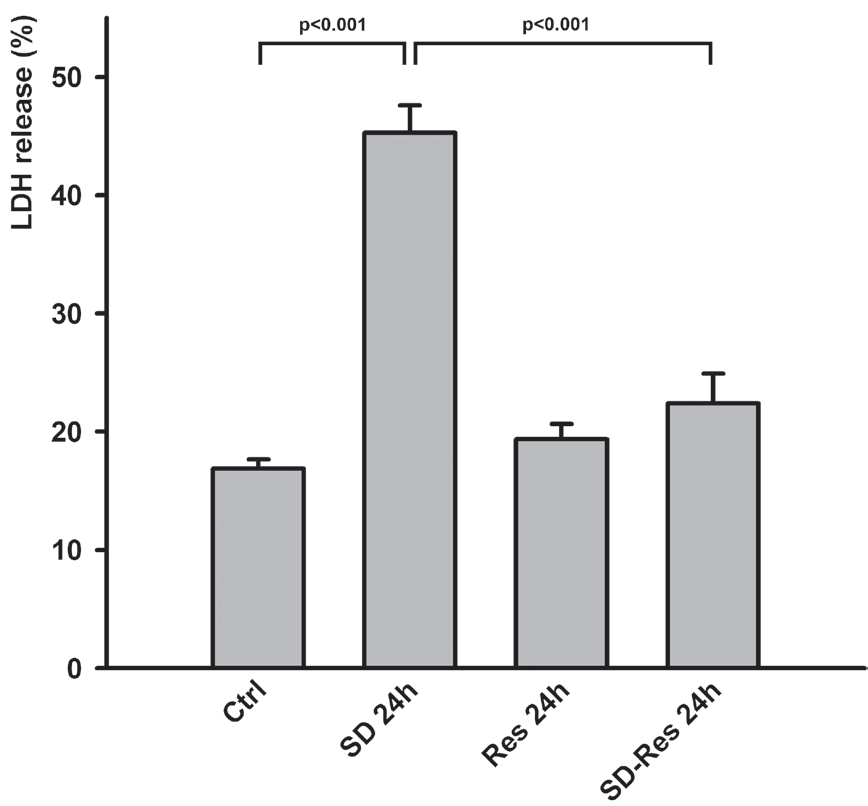
200 µM of resveratrol reduced lactate dehydrogenase release after 24 h serum deprivation.

Lactate dehydrogenase release was measured to evaluate whether the inhibition of caspase 3 activation by resveratrol was accompanied by increased cell viability. Cell viability decreased by 24 hour serum deprivation was significantly improved by 200 µM resveratrol treatment (Figure 4).

### The effect of resveratrol on caspase 3 activity involves p38 kinase pathway

**Figure 5:**
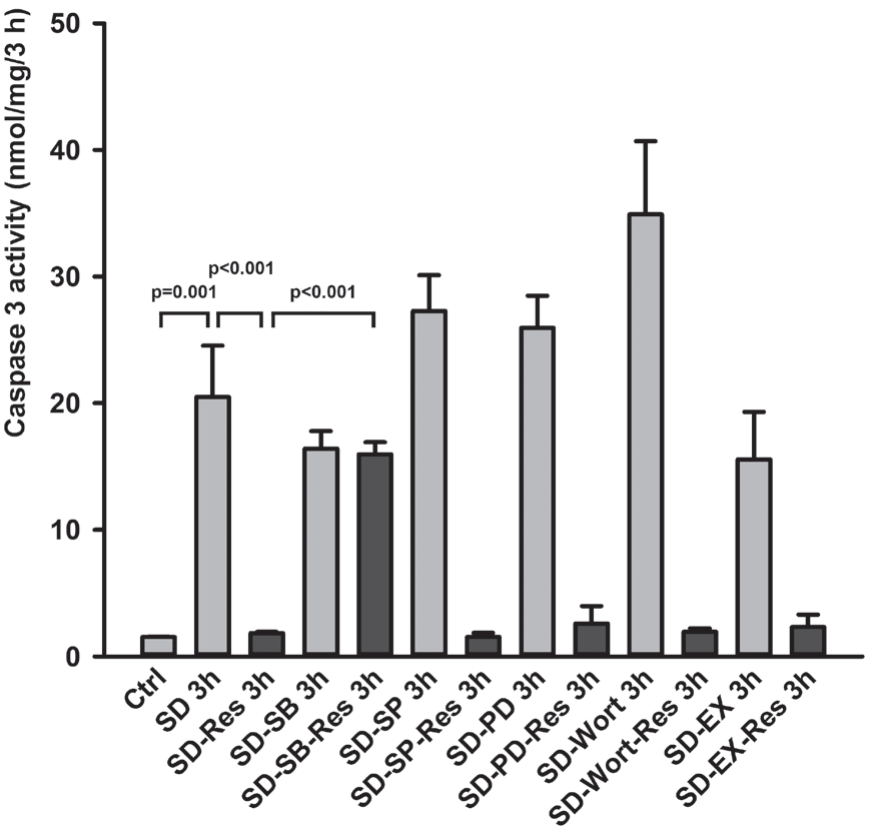
The effect of 50 µM SB202190 (p38 MAPK inhibitor), 50 µM SP600125 (JNK inhibitor), 50 µM PD184352 (ERK inhibitor), 10 µM wortmannin (PI3 kinase inhibitor), and 5 µM EX-527 (SIRT-1 inhibitor) on 3-h serum deprivation-induced caspase 3 activation and the protective action of 200 µM resveratrol. Only p38 MAPK inhibitor SB202190 abolished the effect of resveratrol on caspase activation.

In order to investigate the signaling cascades involved in the protective effects of resveratrol, we carried out experiments in the presence of specific inhibitors of p38 (50 µM SB202190), JNK (50 µM SP600125), ERK (50 µM PD184352), PI3K (10 µM wortmannin) kinase pathways, and SIRT1 (5 µM EX-527). Among them, only p38 MAPK inhibitor SB 202190 decreased the protective effect of resveratrol on caspase 3 activation (Figure 5).

### The role of oxidative stress in the effect of resveratrol

**Figure 6:**
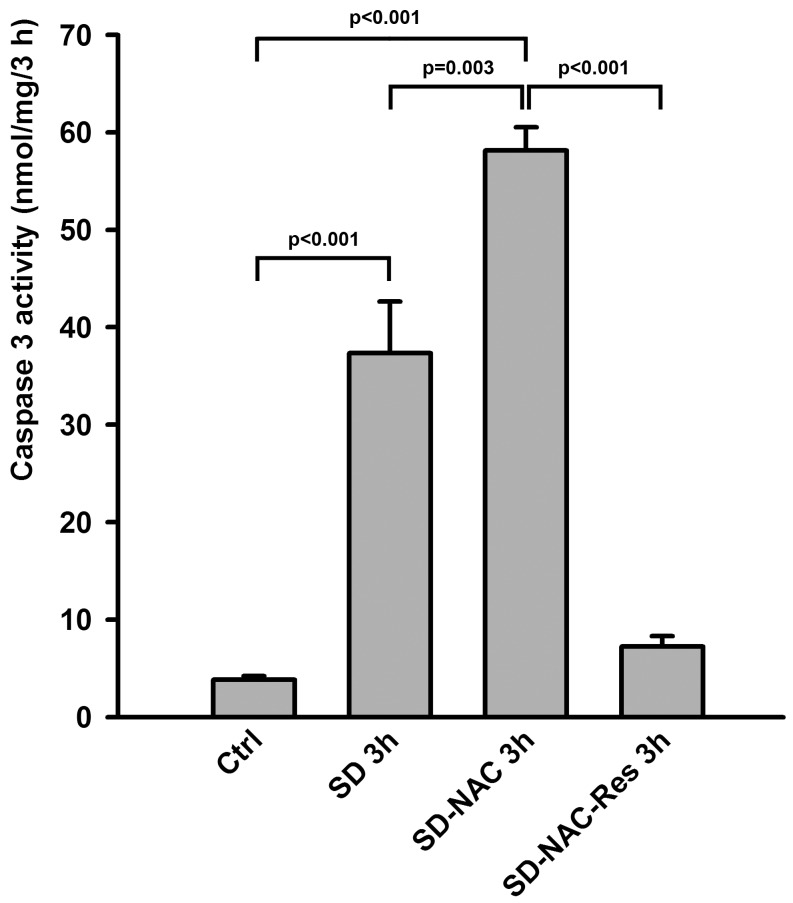
Five mM of N-acetylcysteine (NAC) exacerbated serum deprivation-induced caspase 3 activation, but 200 µM of resveratrol prevented their combined effect.

Considering that p38 kinase pathway is activated by mild intracellular stress (13) and pro- and antioxidant properties of resveratrol had been previously described (14), we hypothesized that reactive oxygen species generation could be involved in caspase 3 activation induced by serum deprivation and/or the protective effect of resveratrol. To clarify if the antioxidant property of resveratrol may play a key role in its cytoprotective effect, we investigated the effect of 5 mM N-acetylcysteine, a well-known antioxidant agent, on caspase 3 activation. Contrary to our expectations, it did not prevent caspase 3 activation but exacerbated it. However, 200 µM resveratrol abolished the combined effect of serum deprivation and N-acetylcysteine on caspase 3 activation (Figure 6).

## Discussion

### Cytoprotective effect of resveratrol

Resveratrol prevented serum deprivation-induced caspase 3 activation in primary fibroblasts and increased their viability. These results are in line with those of previous studies performed on non-transformed cells using various toxic insults (15,16). In this study, cytoprotective effect of resveratrol was considerable, in 100-200 µM concentration range, which is similar to another study (17). However, some recent studies observed lower concentrations, in the 10-20 µM range to be efficient as well (9,16). The effective dose probably depends on the cell type and the intensity of the damaging insult used. The concentration found to be effective in the present study is considerably higher than the concentration that can be obtained from dietary sources, suggesting the need for resveratrol supplementation. Furthermore, resveratrol can serve as a lead compound for research of more potent cytoprotective medications.

To the best of our knowledge this is the first report demonstrating that resveratrol abolishes the already elevated caspase 3 activity induced by serum deprivation, suggesting its rescue effect. Resveratrol was found to prevent and improve cardiac function in cardiac fibroblasts (18,19) and to play a neuroprotective role in neurotoxic injury (20). However, our results showed that it is a promising cytoprotective agent which should be explored not only for prevention of age-related degenerative disorders, but also in the early treatment of degeneration following an acute insult.

### Probable mechanism of resveratrol action

It has already been suggested that several kinase pathways have a role in the cytoprotective effects of resveratrol. Cytoprotective functions of resveratrol were associated with the activation of PI3-kinase/Akt (21,22), p38 MAPK/JNK/ERK (23,24) signaling, and molecular pathways involving SIRT1 (9), an NAD dependent histone deacetylase. Our present findings indicate that the most critical signaling pathway in the protective effect of resveratrol against serum deprivation-induced caspase 3 activation is the activation of p38. The reports about the effects of resveratrol on p38 kinase pathway are rather contradictory. It was shown that through inhibition of p38 pathway resveratrol suppresses macrophage and vascular smooth muscle cell apoptosis (17,23). On the other hand, it exerted protective effect in H9c2 embryonic rat heart derived cells by up-regulating the p38 MAPK signaling (25). It was also shown to inhibit the proliferation of human primary fibroblasts and enhance their entry to senescence in p38 dependent manner (26). Therefore, p38 kinase seems to have a dual role as a regulator of cell fate, mediating either survival or death. Adams et al (27) reported that the specific function of p38 MAPKs in apoptosis depended on the cell type, stimuli, and/or p38 isoform. In accordance with their findings, we showed that p38 MAPK had a cytoprotective rather than proapoptotic role.

Several articles discuss antioxidant properties of resveratrol as the cause of its cytoprotective effect (14,16). Considering that N-acetylcysteine exacerbated rather than prevented serum deprivation-induced caspase activation and resveratrol abolished their combined effect, antioxidant properties cannot explain its protective action. Previous articles reported similar effect of N-acetylcysteine, concluding that the elevated glutathione level can inhibit NF-κB induced transcription of inhibitor of apoptosis protein, which can explain its potentiating effect on caspase activation (28,29). Since several previous reports demonstrated not only antioxidant but prooxidant characteristics of resveratrol (14,30), the latter might be involved in the activation of p38 MAPK and reduction of caspase 3 activation. A previous study suggested that the prooxidant activity of resveratrol was responsible for its inhibitory effect on apoptosis by creating an intracellular milieu non-permissive for caspase activation (30). These findings are in line with our results, which also indicate the role of p38 kinase in the protective effect of resveratrol and activation of this pathway by mild intracellular stress (13). However, the effect of resveratrol on oxidative state of cells requires further research.

Activation of p38 was also connected to increase in autophagic flux. This process is involved in the degradation of misfolded proteins or damaged organelles, such as depolarized mitochondria, which can prevent the release of proapoptotic mediators and the consequent caspase activation (25). Accordingly, two recent papers reported that resveratrol improved autophagic flux and prevented caspase cleavage in H9c2 rat cardiomyoblast cells (25,31). Similarly to our results, the protective effect of resveratrol depended on p38 MAPK activity (25). Based on these data, we can hypothesize that the effect of resveratrol on caspase 3 activation and cell survival might be connected with its prooxidant property, which may enhance autophagic flux via p38 activation.

A major limitation of this study is its *in vitro* nature, which is why further translational experiments are required to analyze the cytoprotective effect of resveratrol. In conclusion, we demonstrated the p38 MAPK signaling pathway-dependent cytoprotective effect of resveratrol against serum deprivation induced caspase 3 activation in primary fibroblasts. Also, resveratrol exhibited a rescue effect and reduced the already up-regulated caspase 3 activity. This finding may contribute to the research of drugs used for prevention and treatment of age-related disorders.
